# Effects of Long-Term Intervention with Losartan, Aspirin and Atorvastatin on Vascular Remodeling in Juvenile Spontaneously Hypertensive Rats

**DOI:** 10.3390/molecules28041844

**Published:** 2023-02-15

**Authors:** Qi Liu, Shuai Dong, Xue Zhou, Yubo Zhao, Bin Dong, Jing Shen, Kang Yang, Linsen Li, Dan Zhu

**Affiliations:** 1 School of Pharmacy, Minzu University of China, Beijing 100081, China; 2 Chengdu Institute of Biology, Chinese Academy of Sciences, Chengdu 610041, China; 3 Key Laboratory of Carcinogenesis and Translational Research (Ministry of Education/Beijing), Core Laboratory, Peking University Cancer Hospital & Institute, Beijing 100142, China

**Keywords:** juvenile SHR, vascular remodeling, losartan, aspirin, atorvastatin

## Abstract

Hypertension in adolescents is associated with adverse cardiac and vascular events. In addition to lowering blood pressure, it is not clear whether pharmacological therapy in early life can improve vascular remodeling. This study aimed to evaluate the effects of long-term administration of losartan, aspirin, and atorvastatin on vascular remodeling in juvenile spontaneously hypertensive rats (SHRs). Losartan, aspirin, and atorvastatin were administered via gavage at doses of 20, 10, and 10 mg/kg/day, respectively, on SHRs aged 6–22 weeks. Paraffin sections of the blood vessels were stained with hematoxylin-eosin (H&E) and Sirius Red to evaluate the changes in the vascular structure and the accumulation of different types of collagen. The plasma levels of renin, angiotensin II (Ang II), aldosterone (ALD), endothelin-1 (ET-1), interleukin-6 (IL-6), and neutrophil elastase (NE) were determined using ELISA kits. After the 16-week treatment with losartan, aspirin, and atorvastatin, the wall thickness of the thoracic aorta and carotid artery decreased. The integrity of the elastic fibers in the tunica media was maintained in an orderly manner, and collagen deposition in the adventitia was retarded. The plasma levels of renin, ALD, ET-1, IL-6, and NE in the SHRs also decreased. These findings suggest that losartan, aspirin, and atorvastatin could improve vascular remodeling beyond their antihypertensive, anti-inflammatory, and lipid-lowering effects. Many aspects of the protection provided by pharmacological therapy are important for the prevention of cardiovascular diseases in adults and older adults.

## 1. Introduction

Hypertension in childhood and adolescence is associated with adverse cardiac changes and vascular damage, which are consequently associated with premature cardiovascular diseases in adulthood [1]. Vascular remodeling is one of the major pathological changes in cardiovascular diseases in myriad adolescents [2]. Under normal physiological conditions, vascular remodeling is an adaptive process that responds to hemodynamic alterations and maintains blood pressure stability. However, permanent vascular remodeling aggravates the narrowing of the vascular lumen and the thickening of the vessel wall [3]. In addition, these unfavorable structural changes contribute to the development of greater arterial stiffness, accompanied by changes in the structure and composition of elastic fibers and collagen [4,5,6]. Moreover, changes in the structure and functional abnormalities of the conduit arteries may lead to the maintenance of hypertension and impairment of the heart, brain, and kidneys, as well as other secondary adverse events [3]. In fact, the characteristic remodeling associated with hypertension is difficult to reverse [3]. For example, layered elastic laminae are produced only from the fetus to early childhood. When the elastic laminae are formed, the genetic program required to produce elastic fibers is permanently silenced [7]. Therefore, early treatment of hypertension in children and adolescents is of paramount importance for the prevention of premature cardiovascular diseases. In addition to lowering blood pressure, therapeutic strategies targeting the vascular structure and function should be seriously considered.

Pharmacological therapy is necessary for children and adolescents who remain hypertensive despite compliance with lifestyle modifications [8]. Losartan, aspirin, and atorvastatin are first-line drugs for cardiovascular diseases. Patients with cardiovascular diseases, including children with hypertension, usually take these three drugs for long-term medication. So, it is particularly significant to study their effects on vascular. Losartan not only significantly reduces the blood pressure of hypertensive patients but also improves aortic stiffness [9,10,11,12]. Low-dose aspirin is recommended by various guidelines for the primary prevention of cardiovascular disease [13,14,15,16], Aspirin can suppress platelet activation and aggregation and is beneficial to reduce aortic stiffness in hypertensive patients [17,18,19]. Atorvastatin exerts beneficial cardiovascular effects supported by experimental and clinical evidence [20,21]. It is worth noting that atorvastatin can improve the arteriosclerosis associated with many diseases, such as hypertension, hyperlipidemia, long-term kidney disease, and type 2 diabetes [22,23,24,25].

Although some studies have shown that these drugs improve vascular structure and function in vivo and in vitro models [26,27,28], more evidence depending on lower doses and various animal models is still worth exploring.

Spontaneously hypertensive rats (SHRs) are widely used as animal models for essential hypertension and vascular lesions. From adolescence to adulthood, the blood pressure of SHRs gradually increases, and the arteries remodeling including vascular wall thickening and wall-to-lumen increase, which have also been observed in patients with hypertension [29]. Therefore, this study administrated SHRs aged from 6 to 22 weeks to explore the effects of long-term administration of losartan, aspirin, and atorvastatin on the vascular structure.

## 2. Results

### 2.1. Effects of Losartan, Aspirin, and Atorvastatin on the Arterial Vessel Structure

#### 2.1.1. Histological Observation of the Thoracic Aorta

**Figure 1:**
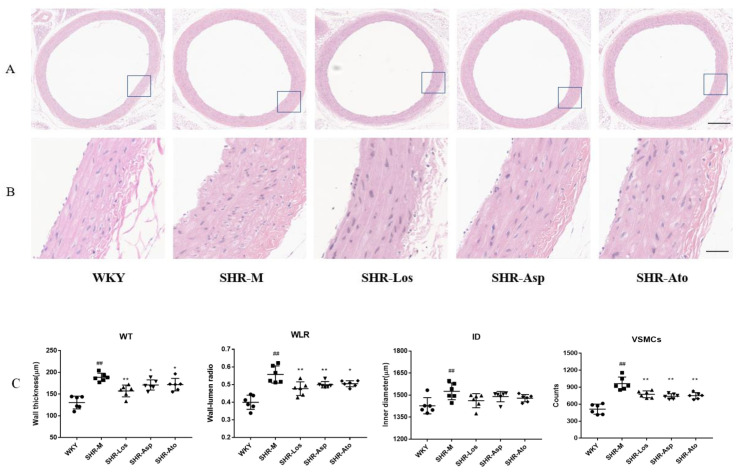
The histological morphological changes in thoracic aorta in each group (22 weeks rats). (**A**) H&E stain in cross section of thoracic aortas. (scale bar = 300 μm); (**B**) magnified images of (**A**) (scale bar = 50 μm); (**C**) wall thickness (WT), wall-to-lumen ratio (WLR), inner diameter (ID) and number of VSMCs (VSMC) in tunica media, respectively. The data are presented as the mean ± SD. *n* = 6, **^##^** *p* < 0.01 vs. WKY; * *p* < 0.05, ** *p* < 0.01 vs. SHR-M.

Compared with those of the Wistar Kyoto rats (WKY) group, the thoracic aortas of the SHR-Model (SHR-M) group showed a significant increase in the wall thickness (WT), wall-to-lumen ratio (WLR), and inner diameter (ID) (Figure 1A); simultaneously, the topical vessel tunica intima thickened. The vascular smooth muscle cells (VSMCs) in the WKY group were characterized by a thin structure and a long spindle shape and were regularly aligned as concentric circles. Meanwhile, the VSMCs in the SHR-M group were unregularly arranged, and the morphology of some VSMCs changed, with a double nucleus (Figure 1B). The number of VSMCs increased significantly in the SHR-M group compared with that in the WKY group (*p* < 0.01). The wall of the thoracic aortas was significantly thinner; the WLR of the thoracic aortas was significantly lower (*p* < 0.05); the total number of VSMCs in tunica media was significantly smaller (*p* < 0.05); and the arrangement of the VSMCs was more regular in the SHR-Losartan (SHR-Los), SHR-Aspirin (SHR-Asp), and SHR-Atorvastatin (SHR-Ato) groups than in the SHR-M groups (Shown in Figure 1).

#### 2.1.2. Histological Observation of the Carotid Artery

**Figure 2:**
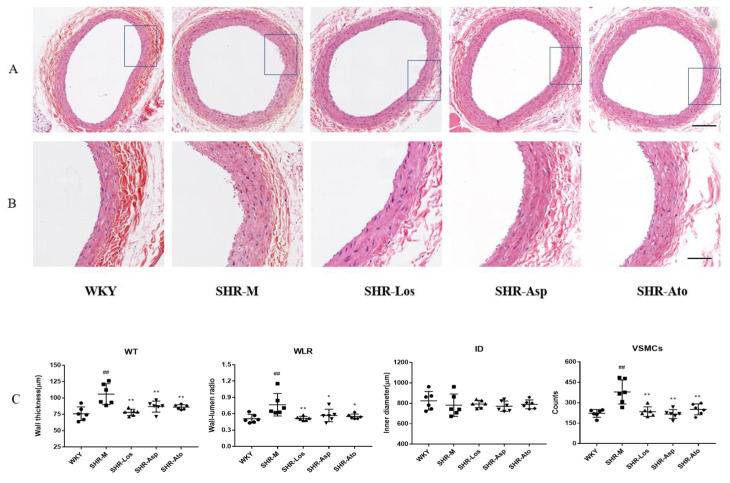
The histological morphological changes in carotid aorta in each group (22 weeks rats). (**A**) H&E stain in cross section of carotid aortas. (scale bar = 200 μm); (**B**) magnified images of (**A**) (scale bar = 100 μm); (**C**) wall thickness (WT), wall-to-lumen ratio (WLR), inner diameter (ID), and number of VSMCs (VSMC) in tunica media, respectively. The data are presented as the mean ± SD. *n* = 6, **^##^** *p* < 0.01 vs. WKY; * *p* < 0.05, ** *p* < 0.01 vs. SHR-M.

As shown in Figure 2, the changes in the WT (tunica media), WLR, and VSMCs parameters in the carotid arteries between the WKY and SHR-M groups were similar to those in the thoracic aorta. The WT, WLR, and total number of VSMCs in tunica media in the SHR-Los, SHR-Asp, and SHR-Ato groups markedly decreased compared with those in the SHR-M group (*p* < 0.05). The ID of the WKY group was larger than that of the SHR-M group; however, there was no significant difference between the groups.

### 2.2. Effects of Losartan, Aspirin, and Atorvastatin on the Distribution of Elastin

**Figure 3:**
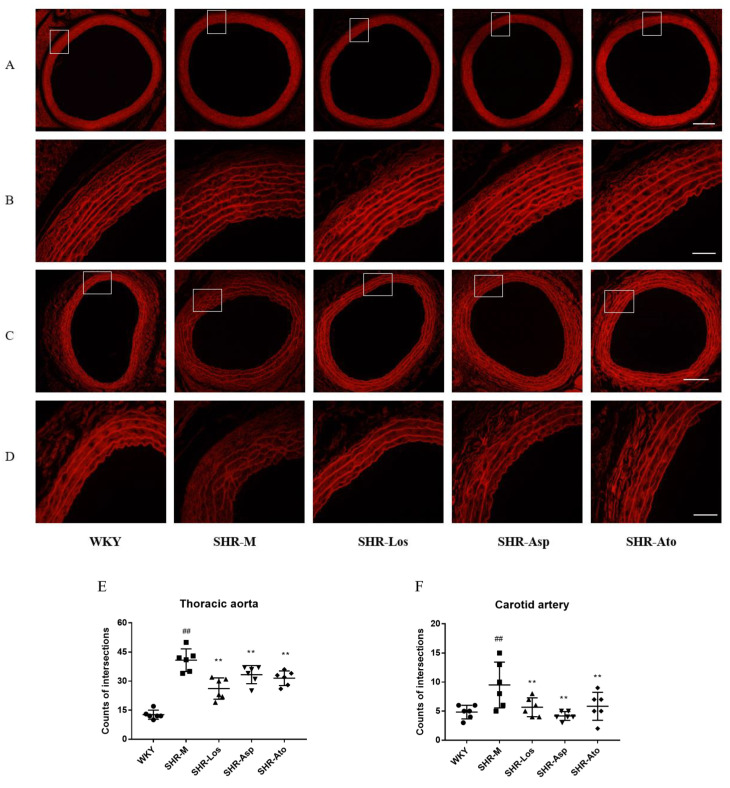
Thoracic aortas (**A**,**B**) or carotid arteries (**C**,**D**) elastin by H&E stain under fluorescence microscope showed intense red autofluorescence (22 weeks rats). (**B**,**D**) are enlarged views of (**A**,**C**), respectively (**A**: scale bar = 300 μm; **B**: scale bar = 50 μm; **C**: scale bar = 200 μm; **D**: scale bar = 50 μm); (**E**) counts of link across elastin fiber layers in thoracic aortas; (**F**) counts of link across elastin fiber layers in carotid arteries. The data are presented as the mean ± SD. *n* = 6, **^##^** *p* < 0.01 vs. WKY; ** *p* < 0.01 vs. SHR-M.

As shown in Figure 3, the elastic fiber layer exhibited strong red autofluorescence. The elastic fiber layers of the thoracic aortas (A and B) and carotid arteries (C and D) were arranged in an orderly manner in the WKY group, with few fractures, and there were few links across the layers. The elastin fiber layers in the SHR-M group were arranged disorderly, with more fractures, significantly more links across the layers, and weaker fluorescence, compared with those in the WKY group. The elastin fiber layers in the SHR-Los, SHR-Asp, and SHR-Ato groups were arranged in a more orderly fashion, with fewer fractures and links across layers, than those in the SHR-M group (*p* < 0.01).

### 2.3. Distribution of Collagen Fibers

#### 2.3.1. Thoracic Aorta

**Figure 4:**
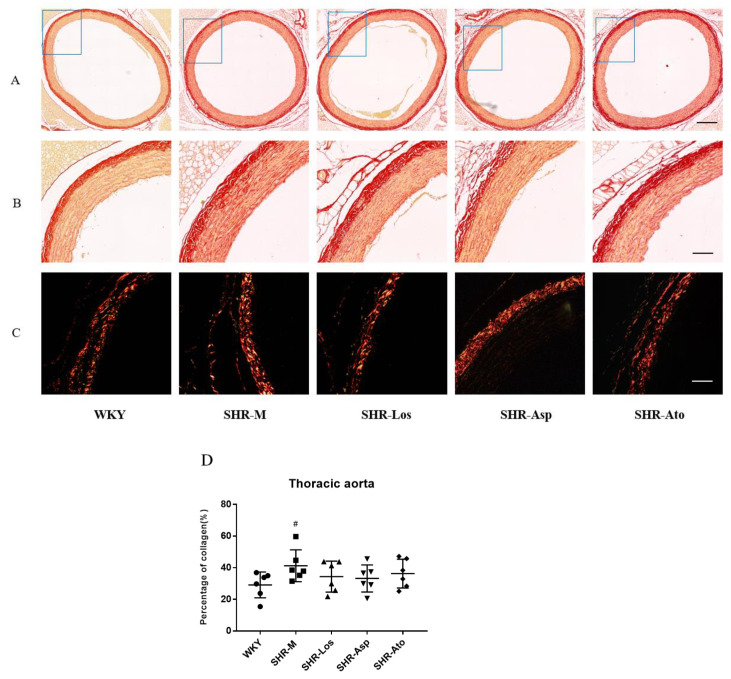
The distribution of collagen fibers in cross sections of thoracic aortas (22 weeks rats). (**A**) Sirius Red stain of collagen fibers under optical microscope (scale bar = 300 μm); (**B**) magnified images of (**A**) (scale bar = 100 μm); (**C**) Sirius Red stain of collagen I and collagen III under polarized light microscopy, collagen I was bright red or strong orange–red, and collagen III was green (scale bar = 100 μm); (**D**) the percentage of collagen fibers in thoracic aortas. The data are presented as the mean ± SD. *n* = 6, **^#^** *p* < 0.05, vs. WKY.

As shown in Figure 4A,B, collagen fibers stained with Sirius Red appeared red under a bright field microscope, while the adventitia appeared high-intensity red. The proportion of collagen fibers in the tunica media of the SHR-M group increased significantly compared with that of the WKY group (*p* < 0.05); the proportion in the three treatment groups decreased compared with that in the SHR-M group. Figure 4C demonstrates the different types of collagen fibers in the vascular wall under a polarized light microscope. Collagen I appeared strongly orange–red or bright red, while collagen III appeared green. There was no significant difference in the proportions of collagen I and III in each group.

#### 2.3.2. Carotid Artery

**Figure 5:**
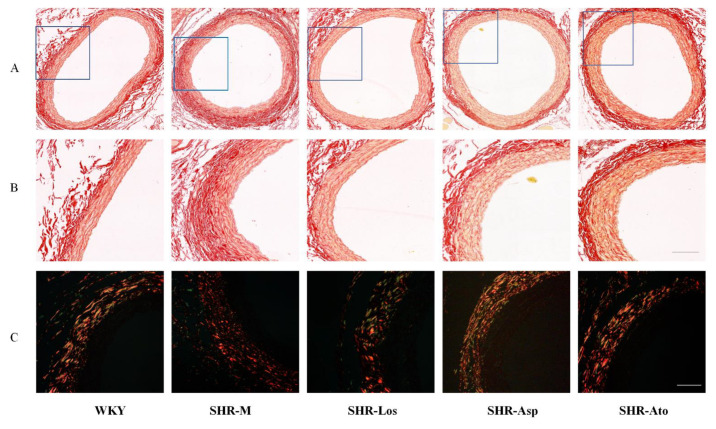

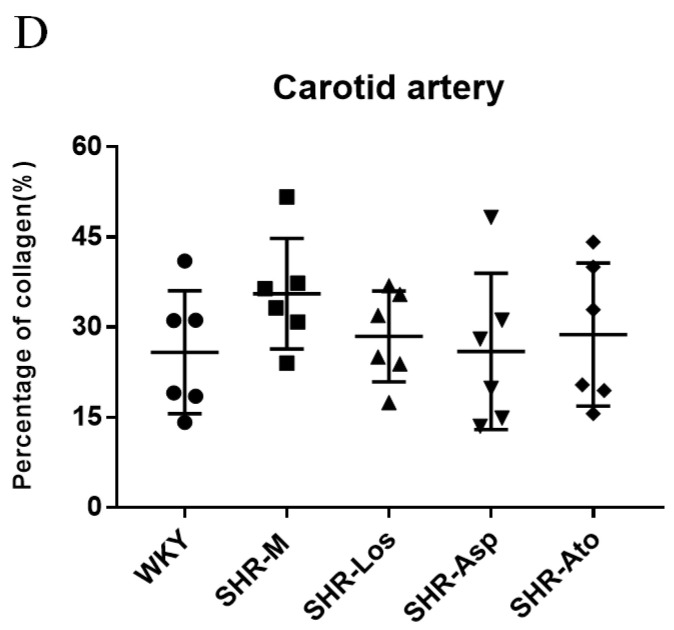
The distribution of collagen in cross sections of carotid arteries (22 weeks rats). (**A**) Sirius Red stain of collagen under optical microscope (scale bar = 200 μm); (**B**) magnified images of (**A**) (scale bar = 100 μm); (**C**) Sirius Red stain of collagen I and collagen III under polarized light microscopy, collagen I was bright red or strong orange–red, and collagen III was green (scale bar = 100 μm); (**D**) the percentage of collagen fibers in carotid arteries. The data are presented as the mean ± SD. *n* = 6.

Similar to the images shown in Figure 4, those shown in Figure 5A,B were obtained under a bright field microscope, and those shown in Figure 5C were collected under a polarized light microscope. The proportion of collagen fibers in the SHR-M group was higher than that in the WKY group, while the proportion in the SHR-Los, SHR-Asp, and SHR-Ato groups was lower than that in the SHR-M group (Figure 5D). As illustrated in Figure 5C, the proportion of collagen III (green) in the SHR-M group was lower than that in the WKY group. The proportion of collagen III in the three treatment groups was higher than that in the SHR-M group.

### 2.4. Effects of Losartan, Aspirin, and Atorvastatin on the Plasma Levels of Renin, Angiotensin II (Ang II), and Aldosterone (ALD)

**Figure 6:**
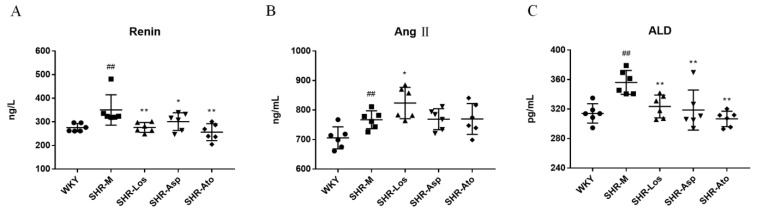
Effects of losartan, aspirin, and atorvastatin on the plasma contents of renin (**A**), Ang II (**B**), and ALD (**C**) in 22 weeks rats. The data are presented as the mean ± SD. *n* = 6, **^##^** *p* < 0.01 vs. WKY; * *p* < 0.05, ** *p* < 0.01 vs. SHR-M.

As shown in Figure 6, the plasma levels of renin, Ang II, and ALD in the SHR-M group were significantly higher than those in the WKY group (*p* < 0.01). Compared with those in the SHR-M group, the renin and ALD levels were obviously reduced in the three treatment groups (*p* < 0.05). The Ang II level in the SHR-Asp and SHR-Ato groups was slightly lower than that in the SHR-M group. Meanwhile, the Ang II level in the SHR-Los group was higher than that in the SHR-M group (*p* < 0.05).

### 2.5. Effects of Losartan, Aspirin, and Atorvastatin on the Plasma Levels of Endothelin-1 (ET-1), Neutrophil Elastase (NE), and Interleukin-6 (IL-6)

**Figure 7:**
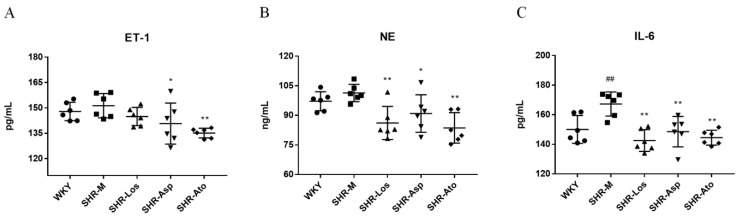
Effects of losartan, aspirin, and atorvastatin on the plasma contents of ET-1 (**A**), NE (**B**), and IL-6 (**C**) in 22 weeks rats. The data are presented as the mean ± SD. *n* = 6, **^##^** *p* < 0.01 vs. control (WKY); * *p* < 0.05, ** *p* < 0.01 vs. SHR-M group.

As shown in Figure 7A, the plasma level of ET-1 was the highest in the SHR-M group; the level in the SHR-Asp and SHR-Ato groups was significantly lower than that in the SHR-M group (*p* < 0.05). As exhibited in Figure 7B,C, compared with those in the WKY group, the plasma levels of NE and IL-6 in the SHR-M group increased. As shown in Figure 7B, losartan, aspirin, and atorvastatin markedly reduced the NE levels (*p* < 0.05). Meanwhile, the IL-6 level in the SHR-Los, SHR-Asp, and SHR-Ato groups was significantly lower than that in the SHR-M group (*p* < 0.01).

## 3. Discussion

Juvenile hypertension tends to lead to adverse cardiovascular events in adulthood. Vascular remodeling occurs in youth and adults with hypertension and is associated with the development of hypertension-related complications. SHRs have been used as animal models of human essential hypertension in more than 17,000 studies since they were bred from WKY rats by Okamoto in 1963 [30,31]. Currently, this strain rat is far from being a model of hypertension but is a more extensive model of arteriosclerosis, cardiac hypertrophy, and cerebrovascular diseases. The occurrence and development of these diseases are associated with vascular remodeling. If hypertension is not managed in adolescents, it can continue into adulthood and old age. Therefore, in our study, five-week-old SHRs whose blood pressure rises gradually were selected as the animal model for adolescents hypertension [32], and losartan, aspirin, and atorvastatin were administered at the age of 6 weeks. After long-term intervention, the effects on vascular remodeling were observed at the age of 22 weeks.

Blood pressure represents hemodynamic conditions and is one of the most commonly monitored clinical parameters for therapeutic decisions. In our study, losartan can significantly reduce systolic blood pressure (SBP) and diastolic blood pressure (DBP), but long-term administration of aspirin and atorvastatin has no significant effect on systolic and diastolic blood pressure in spontaneously hypertensive rats (the blood pressure of SHR rats aged 6–18 weeks (dosing 0–12 weeks) has been published in Dong et al., 2023, Molecules [33], and blood pressure of 22 weeks was included in the Supplementary Materials). Blood pressure regulation is a complex physiologic function that depends on the integrated actions of multiple cardiovascular, renal, neural, endocrine, and local tissue control systems [34]. One of the reasons may be that only losartan has an effect on the gut microbiota, while the other two did not [33]. Although aspirin and atorvastatin can improve vascular remodeling, that is not enough to bring an obvious blood pressure change. Just as aging of vascular in the elderly is accompanied by the appearance of arterial stiffness, only a proportion of them also suffer from hypertension. Nonetheless, our research found that long-term administration of losartan, aspirin, and atorvastatin in the early stages of hypertension may improve vascular remodeling and reduce arteriosclerosis in hypertensive rats, although aspirin and atorvastatin have no strong antihypertensive effects.

Vascular remodeling is characterized by VSMC proliferation and hypertrophy, vessel wall thickening, and increased WLR. Herein, the thoracic aortas (large artery) and carotid arteries (middle artery) of rats at the age of 22 weeks were observed using blood vessel parameters. ID parameter of thoracic aortas in SHR-M was larger than that in WKY, indicating outward remodeling, but carotid arteries in SHR-M did not show obvious outward or inward inclination. The WT, WLR, and number of VSMCs in the three treatment groups were significantly lower than those in the SHR-M group; these parameters were the lowest in the SHR-Los group. These findings indicate that losartan, aspirin, and atorvastatin could improve both large and middle vascular remodeling and that lowering the blood pressure of losartan yields the best effect.

The noncellular components of the vascular wall determine vessel elasticity and stiffness. Elastin and collagen fibers constitute the primary components of the arteries. In healthy and young rats, elastic fibers are in the medial layer of the conduit arteries and arranged in concentric fenestrated elastic lamellae. However, elastin fibers are prone to fragmentation, and their orderly arrangement gradually disappears in cases of arterial stiffness and hypertension [35]. The points across the elastic fiber layers reflect the rearrangement. Our analyses demonstrated that the numbers across the elastic fiber layers in the SHR-M group increased, and the autofluorescence of the elastic fibers in some SHR-M rats was weak, indicating that the elastin content decreased. However, the elastic fibers both in the thoracic aortas and carotid arteries of the SHR-Los, SHR-Asp, and SHR-Ato groups were arranged in more order than those of the SHR-M group. These results indicate that losartan, aspirin, and atorvastatin alleviated the fracture and degeneration of elastic fibers, which is beneficial for maintaining vascular elasticity.

The degeneration of elastic fibers is accompanied by the accumulation of collagen, resulting in arterial stiffness and vascular wall thickening [36]. Our experiment showed that the proportion of collagen in the aortas and carotid arteries of the SHR-Los, SHR-Asp, and SHR-Ato groups was lower than that of the SHR-M group, implying that the stiffening of the blood vessels was retarded after treatment. The collagen of the vascular wall is mainly types I and III collagen. Collagen I is thicker and harder, which is related to the rigidity of the arterial wall. Collagen III is thinner, more ductile, and elastic, which is related to the compliance of the arteries [37]. We found that there was more collagen III proportion in the carotid arteries of the SHR-Los, SHR-Asp, and SHR-Ato groups than in those of the SHR-M group, which reflected better compliance of the middle arteries in the three treatment groups.

In addition to VSMCs, endothelial cells are involved in vascular remodeling. ET-1 is primarily secreted by endothelial cells. It interacts with ET-1 receptors located in VSMCs to induce continuous local vasoconstriction [38]. The levels of ET-1 increase in many hypertension cases. ET-1 also plays a pro-inflammatory role and induces vascular-related injury. In addition, it activates the renin–angiotensin–aldosterone system (RAAS) and sympathetic nervous system [39]. Herein, the plasma level of ET-1 in the SHR-Los, SHR-Asp, and SHR-Ato groups was lower than that in the SHR-M group, which demonstrated that the three drugs improved the function of vascular endothelial cells and end-organs in the SHRs to some extent. The ET-1 level did not significantly decrease in the SHR-Los group compared with that in the SHR-M group, suggesting that the inhibition of vasoconstriction in the SHR-Los group was mainly attributed to the blockade of the Ang II type 1 receptor.

The RAAS is one of the most important hormone systems that regulate blood pressure, body fluid volume, and sodium–potassium balance. Its disorder plays an important role in the development and maintenance of hypertension [40]. An increase in Ang II production activates the RAAS and yields a strong vasoconstrictive effect. Ang II is associated with vascular remodeling because it mediates VSMC proliferation and extracellular matrix synthesis. In addition, it can lead to endothelial cell damage through inflammation and blood coagulation, which promote vascular remodeling [41]. In this experiment, the renin and ALD levels decreased in the three treatment groups. The Ang II level of the SHR-Asp and SHR-Ato groups was similar to that of the SHR-M group. However, it was higher in the SHR-Los group than in the SHR-M group. This might be a compensatory increase attributed to the blockage of the Ang II type 1 receptor by losartan. Interestingly, the increase in the Ang II level was not consistent with the decrease in the renin and ALD levels in the RAAS after losartan administration. The RAAS includes two pathways: the angiotensin-converting enzyme I-angiotensin II type 1 receptor and the angiotensin-converting enzyme II-Mas receptor axes [42]. We speculated that angiotensin-converting enzyme I might be expressed at higher levels in SHRs and the highest levels in SHR-Los rats. This could explain why aspirin and atorvastatin improved vascular remodeling, although they did not have a strong antihypertensive effect.

Accordingly, the proliferation and phenotypic transformation of smooth muscle cells or imbalance in the RAAS ultimately affects the changes in blood pressure and vascular remodeling, which are related to inflammation. It is well known that hypertension and cardiovascular diseases are accompanied by a state of long-term low-level inflammation [43]. Therefore, we evaluated two inflammatory factors implicated in cardiovascular diseases. IL-6 plays an important role in inflammation and can regulate the development of a variety of diseases, including hypertension and other cardiovascular diseases [44]. The blood levels of IL-6 in patients with hypertension are higher, and an increase in the levels of IL-6 is related to vasoconstriction and remodeling [45,46,47,48]. NE is primarily passively released or actively secreted from neutrophils. Plasma NE plays an important role in both acute and chronic inflammatory diseases [49]. Previous studies have proven that increases in the NE level are related to endothelial injury, arterial stiffness, elevated blood pressure (formerly known as “pre-hypertension”), and hypertension [50]. In our study, losartan, aspirin, and atorvastatin reduced the plasma levels of IL-6 and NE in the SHRs, suggesting that the three drugs achieved the same efficacy in improving vascular remodeling and decreasing inflammatory levels, although via different active mechanisms.

Losartan was reported by several clinical observations to improve arterial stiffness [12,51,52,53,54], other antihypertensive drugs alone or in combination also show similar effects [55], and it seems that this effect is attributed to blood pressure reduction. However, improving arterial stiffness of losartan could not be entirely due to its blood pressure reduction. De Cavanagh et al. find that losartan protects high dietary salt–hypertension rats from vascular oxidative stress, exceeding the benefits of blood pressure reduction, and vascular oxidative stress in salt overload condition is relevant to angiotensin receptor 1 activation [26]. In addition, several studies report losartan attenuates expression of TGF-β activators in a mouse model of Marfan syndrome [56], and improves connective tissue abnormalities in systemic sclerosis [57], which could involve in gene expression of metalloproteinases, fibrillin-1 and COL1A1 in SMAD4 mutations causing Myhre syndrome [58]. The findings with respect to losartan also could explain the prominent effect on improving vascular remodeling in our study.

Basically, aspirin is a cyclooxygenase inhibitor as an anti-inflammatory agent, can block the production of prostaglandins and thromboxanes, and hence inhibit platelet activation and its cascade reactions. It has gradually found several other applications [59], especially primary prevention of cardiovascular events in many counties [60]. After a number of long-term clinical observations, weak evidence for the benefit of cardiovascular disease prevention as well as the potential for harm from bleeding have led to a review of the rational use of aspirin, and a dose of less than 100 mg/d also is taken into account to achieve the best balance between benefit and risk [60]. Therefore, it is more meaningful to study low-dose aspirin, which is effective while reducing other risks. We, therefore, chose a dose of 10 mg/kg/day, at which improvement in vascular remodeling provided data to support the benefits of low-dose aspirin. Although aspirin has been comprehensively used in populations, comparatively, its clinical reports about arterial stiffness are not too many [19,61,62], and none of them involve mechanism explanation. It is taken for granted anti-platelet effect duo to direct correlation of platelet activation with arterial stiffness [63,64,65]. Recently, several studies propose that the effect of aspirin on improving arterial stiffness is independent of anti-platelet. That could involve suppressing HIF-1α/TGF-β1/Smads/Snail signaling pathway [66], and collagen production [67]. However, cyclooxygenase-2 also is the key factor with arterial stiffness [68,69,70,71], so, the effects of aspirin on vascular remodeling are attributed to pleiotropic actions.

Several clinical observations reported the effects of atorvastatin on arterial stiffness in various populations [24,72,73]. Although various statins improve arterial stiffness [22], however, it seems beneficial vascular protection of atorvastatin is at least partly independent of lowing cholesterol. Firstly, atorvastatin improves carotid–femoral pulse wave velocity (PWV) in non-hyperlipidemia patients, including type 2 diabetes [25], coronary artery disease [74], systemic lupus erythematosus [75], chronic kidney disease [76]. Secondly, atorvastatin also decreases C-reactive protein (hsCRP) and osteoprotegerin (OPG) [25], vascular endothelial grow factor (VEGF), and sVCAM-1, as well as increases C3 complement [75]. Atorvastatin could improve vascular remodeling in Ang II induced hypertensive rats via the elimination of oxidative stress and elevating endothelial function [77]. Another study found that the vascular remodeling improvement of atorvastatin is associated with a reduction of pro-inflammatory cytokines levels [27]. One in vitro study shows tumor necrosis factor-alpha (TNF-α) and interleukin-1alpha (IL-1α) induce fibroblast, atorvastatin could inhibit matrix metalloproteinase (MMP)-1, 3, 9 release and collagen degradation [78]. The above findings indicate that atorvastatin exerts pleiotropic effects, and greatly supports our cognition with respect to atorvastatin anti-inflammation.

## 4. Materials and Methods

### 4.1. Drugs and Reagents

Losartan, aspirin, and atorvastatin were purchased from Merck Sharp & Dohme Co., Ltd. (Cramlington, UK), Bayer Healthcare (Beijing, China) Co., Ltd. (Beijing, China), and Pfizer Pharmaceutical Co., Ltd. (New York, NY, USA), respectively. Ang II, renin, ET-1, IL-6, and NE ELISA kits were obtained from Cusabio Technology Co., Ltd. (Wuhan, China). ALD ELISA kits were obtained from Shanghai Enzyme-linked Biotechnology Co., Ltd. (Shanghai, China).

### 4.2. Animal Experiments and Design

Six-week-old male SHRs (130–150 g) and five-week-old male Wistar Kyoto (WKY) rats (130–150 g) were obtained from Charles River, Inc (Beijing, China, SCXK (Jing) 2016-0006). All animals were housed under controlled light (12 h/12 h light–dark cycle), humidity 55% ± 5% and temperature 22.1 °C ± 0.5 °C with free access to water and standard chow. There was one week of adaptive feeding before the formal experiment. All experimental procedures were approved by the Biological and Medical Ethics Committee, Minzu University of China (approval No. ECMU201807).

A total of 32 SHRs were divided into 4 groups: SHR-M (double-distilled water), SHR-Losartan (SHR-Los, 20 mg/kg/day) [79], SHR-Aspirin (SHR-Asp, 10 mg/kg/day) [80], and SHR-Atorvastatin (SHR-Ato, 10 mg/kg/day) [81]. All these drugs were dispersed in double-distilled water and were administrated daily to SHRs via oral gavage for 16 weeks. WKY rats were treated with double-distilled water as normotensive controls.

### 4.3. Histological Morphology of Arterial Vessels

The thoracic aortas and the carotid arteries of rats were dissected and then fixed in 4% paraformaldehyde over 24 h. Fixed tissue was embedded in paraffin, sectioned (3 μm), and stained with H&E. An image acquisition system (Aerio CS2, Leica, Wetzlar, Germany) was used for scanning histological morphology. Wall thickness (WT) and inner diameter (ID) were measured by Image Scope version 12.1.

### 4.4. Vascular Collagen and Elastin

To compare the difference in vascular collagen content between groups, the tissue sections were stained with Sirius Red. Sections were deparaffinized and hydrated to distilled water. Then, sections were placed in Sirius Red solution for 8 min followed by dehydration and mounting. Sirius Red dye is strongly acidic and easy to combine with basic groups in collagen molecules. Collagen fibers were stained a red–orange color and observed by an image acquisition system (Aerio CS2, Leica, Germany). Image Pro Plus version 7.0 was used to calculate the percentage of collagen fibers in tunica media. Sirius Red bonds with collagen fibers, which makes collagen fibers produce obvious birefringence. Under the polarizing microscope (DM2700P, Leica, Germany; polarization angle is 90 degrees), through the characteristics of enhanced birefringence light, type I and III collagen fibers show different colors, and type I collagen fibers show red–orange red; type III collagen fibers are green.

Autofluorescence of elastic fibers in tissue sections stained H&E was recorded with excitation wavelength at 460 nm–550 nm (Olympus 1 × 2 UCB) [32,82].

### 4.5. Assay of Renin, Ang II, ALD, ET-1, NE, and IL-6 Levels in Plasma

All rats were deeply anesthetized using 2% pentobarbital sodium (30 mg/kg) at rats 22 weeks old. Blood was collected from the abdominal aorta into a blood collection tube containing EDTA∙K_2_, then centrifuged at 4000 r/min for 20 min at 4 °C as soon as possible, the plasma was collected and stored at −80 °C, immediately. Commercial ELISA kits were used to measure the level of renin, Ang II, ALD [83], ET-1, NE, and IL-6 in plasma according to their manufacturer’s instructions. ELISA test was completed within 48 h.

### 4.6. Statistical Analyses

Statistical analyses were conducted using SPSS Statistics 19 software. Differences between groups were performed using a one-way analysis of variance (ANOVA), followed by an LSD post hoc test. Comparisons between the 5 groups in this study were presented as mean ± standard deviation (SD). A value of *p* < 0.05 was considered significant.

## 5. Conclusions

This study demonstrated that the administration of losartan, aspirin, and atorvastatin at juvenile ages reduced vascular remodeling in adulthood among the SHRs. The common mechanism involves mitigating the ET-1 and inflammatory marker levels; however, it does not have direct relevance to Ang II. These results also inspire that low dose administration of aspirin or atorvastatin in comprehensive patients suffering cardiovascular diseases could maintain vascular elasticity structure in the premise of ensuring safety

## Data Availability

Not applicable.

## Supplementary Materials

The following supporting information can be downloaded at: https://www.mdpi.com/article/10.3390/molecules28041844/s1, Figure S1: Blood pressure changes after administration in each group.

## Sample Availability

Samples of the compounds are not available from the authors.
