# Supplementary material for: Effects of Long-Term Intervention with Losartan, Aspirin and Atorvastatin on Vascular Remodeling in Juvenile Spontaneously Hypertensive Rats
Source: Molecules. 2023 Feb 15;28(4):1844. doi: 10.3390/molecules28041844 (PMC9965824; doi:10.3390/molecules28041844)
Supplement: Supplementary file 1 [file molecules-28-01844-s001.zip › molecules-2185435-supplementary.pdf]

molecules

MDPI

Supplementary Materials

Effects of Long-Term Intervention with Losartan, Aspirin and Atorvastatin on Vascular Remodeling in Juvenile Spontaneously Hypertensive Rats

Qi Liu [1], Shuai Dong [1], Xue Zhou [2], Yubo Zhao [1], Bin Dong [3], Jing Shen [3], Kang Yang [1], Linsen Li [1,*] and Dan Zhu [1,*]

[1] School of Pharmacy, Minzu University of China, Beijing 100081, China;
[2] Chengdu Institute of Biology, Chinese Academy of Sciences, Chengdu 610041, China
[3] Key Laboratory of Carcinogenesis and Translational Research (Ministry of Education/Beijing), Core Laboratory, Peking University Cancer Hospital & Institute, Beijing 100142, China
* Correspondence: 2008027@muc.edu.cn (L.L.); zhudan@muc.edu.cn (D.Z.); Tel.: +86-158-1089-2058 (D.Z.)

Method

Systolic blood pressure (SBP) and diastolic blood pressure (DBP) were measured by a noninvasive computerized tail-cuff system (Softron BP-2010, Softron Co., Ltd., Tokyo, Japan). To detect the tail artery pulsations and to maintain a stable level, the rats were warmed for 10 min, and placed in a restraining chamber. The whole process of measuring blood pressure was in a quiet environment, and blood pressure was obtained by averaging 7–10 measurements.

Results

Over time, the systolic blood pressure (SBP) of Wistar Kyoto rats (WKY) was maintained at about 140 mmHg, and the diastolic blood pressure (DBP) was maintained at about 100 mmHg. The SBP of SHR rats increased from about 160 mmHg at 6 weeks of age to about 215 mmHg at 22 weeks of age. The SBP and DBP of SHR were significantly higher than those of WKY ($p < 0.01$). After four weeks of a losartan treatment, the SBP and DBP of SHR-Los rats were significantly lower than that in SHR-M ($p < 0.01$). After the atorvastatin and aspirin treatments, the SBP and DBP in SHR-Asp and SHR-Ato rats did not change significantly.

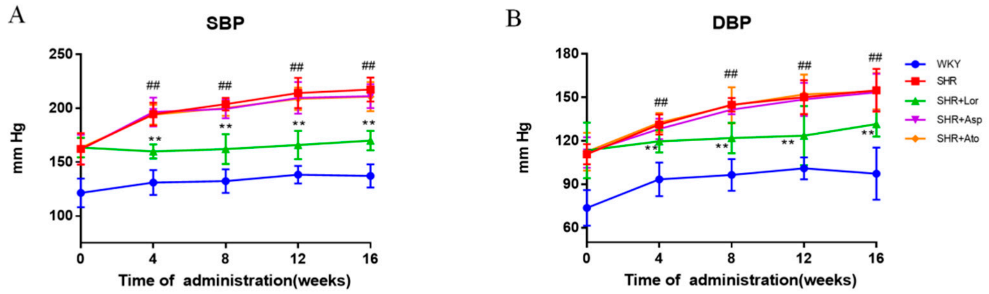

Figure S1. :Blood pressure changes after administration in each group.(age of sacrificed SHR rats: 22 weeks) (A) Systolic blood pressure (SBP); (B) Diastolic blood pressure (DBP). The data are presented as the mean ± SD. *n*=6. Data are expressed as mean ± SD. ##$P < 0.01$ vs WKY, **$P < 0.01$ vs SHR-M
